# How Transcranial Doppler can assess the effect of hyperosmolar therapy and the degree of circulatory compromise in acute brain herniation

**DOI:** 10.1186/2193-1801-2-319

**Published:** 2013-07-15

**Authors:** Hosam Al-Jehani, Mohammad Alkutbi, Mohammad Maleki, Judith Marcoux, Jeanne Teitelbaum

**Affiliations:** Department of Neurology and Neurosurgery, Montreal Neurological Institute and Hospital, 3801 University St., Suite 109, Montreal, QC H3A 2B4 Canada; Neurocritical Care Unit, Montreal Neurological Institute and Hospital, Montreal, QC Canada; Department of Neurosurgery, King Fahad University Hospital, Dammam University, Al-Khobar, Saudi Arabia

**Keywords:** Transcranial doppler, Cerebral circulatory arrest

## Abstract

Patients in acute neurological extremes secondary to refractory intracranial hypertension are challenging because of the complex management options available to them, especially when compounded with signs of brainstem compromise. Objective evidence of cerebral circulatory compromise is often lacking.

We present a case in which an objective evaluation of a cerebral circulatory compromise was documented using transcranial Doppler as well as its resolution with hyperosmolar therapy.

## Background

Mannitol is a widely accepted in therapy for the treatment of intracranial hypertension (ICHT), as it has positive effect on cerebral perfusion pressure (CPP) and cerebral blood flow (CBF) more so in those patients with focal injury (vs. diffuse), those with lesions on the brain imaging, and those in whom the CPP is at or below the auto regulation threshold (Brown et al. 1979;Mendelow et al. 1985;Rosner & Coley 1987;Bratton et al. 2007;Wakai et al. 2007). The mannitol doses reported in these studies ranged from 0.25 to 0.5 gm/kg bolus of 20% mannitol given intravenously over 10 to 15 minutes. In acute herniation, high doses (1.5 g/kg) have been shown to improve patient outcome (Cruz et al. 2001;Cruz et al. 2002;Cruz et al. 2004).

Transcranial Doppler (TCD) has been widely used for the assessment of cerebral blood flow in several clinical conditions (Topcuoglu 2012) including intracranial hypertension. TCD is gaining acceptance as a rapid, portable and reliable method for assessing intracranial hypertension and cerebral blood flow compromise. In their recent study, Tazarourte et al., reported that 50% of TCD’s performed prior to the arrival to the trauma center were abnormal, resulting in administration of hyperosmolar therapy in the ambulance (Tazarourte et al. 2011). Moreover, only patients with abnormal TCD examination required emergency surgery and interestingly those patients in whom the TCD parameter where not improved after the administration of such therapy died within 48 hours (Tazarourte et al. 2011). The case presented in this article illustrates the usefulness of TCD in the monitoring of ICHT leading to herniation and its ability to assess response to therapy.

## Case report

**Figure 1:**
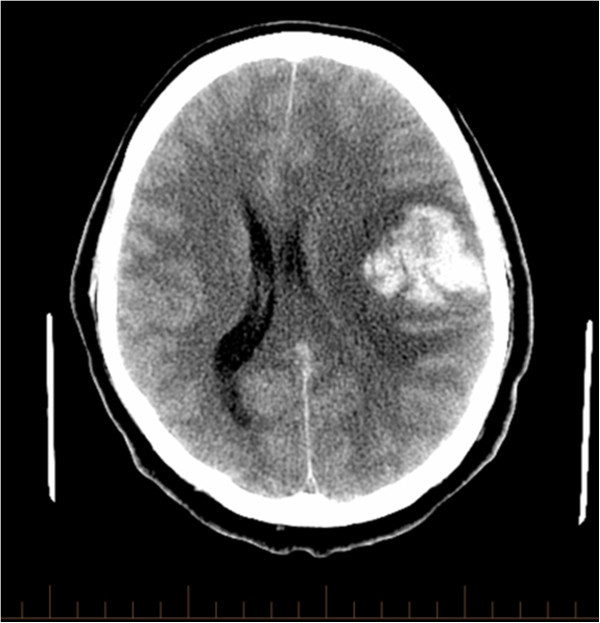
**Non-contrast axial CT scan showing large frontal hemorrhage with peri-lesional edema and evidence of mass effect.**

A 49-year-old right-handed male presented to a local community hospital with headache and progressive right-sided weakness. His past medical history included renal cell carcinoma, in remission for the past 3 years, treated by surgical resection and chemotherapy. The computerized tomography (CT) scan of the head performed on the day of presentation revealed a large hemorrhagic lesion in the left central region of the brain highly suspicious of a tumoral bleed, as well as significant peri-lesional edema, severe midline shift and uncal herniation (Figure 1).

**Figure 2:**
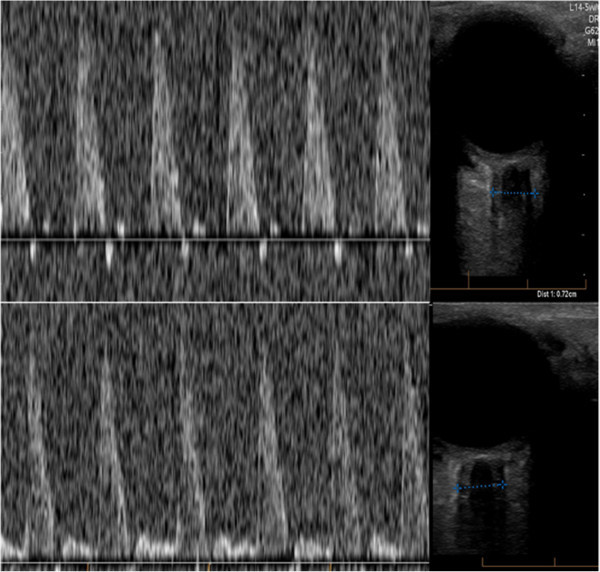
**The upper panel shows the transcranial Doppler of the left middle cerebral artery with a prominent systolic spike with a diastolic descent in the Doppler tracing suggestive of reverberating flow seen in circulatory arrest.** The lower panel shows the transcranial Doppler of the right middle cerebral artery with high resistance circulatory pattern with a high systolic peak and low diastolic velocity suggestive of malignant intracranial hypertension, with a pulsatility index (PI = peak systolic-end diastolic velocities/mean flow velocity) of 2.5. The optic nerve ultrasonography seen on the right side of the figure are corresponding to the side of the middle cerebral artery insonation and were measuring 7.2 mm and 6.4 mm in the left and right optic nerve sheaths, respectively.

Within 2 hours of his arrival, the patient deteriorated, with a drop in Glasgow Coma Scale (GCS) from 15 to 12 prompting urgent intubation prior to transfer to our hospital. Upon arrival in our emergency room, the patient’s neurological examination revealed non-reactive pupils, absent corneal reflexes and extremely abnormal eye movements on oculo-cephalic reflex testing. The patient was hyperventilated and given a 200 mL bolus of 20% mannitol (0.58 g/kg). A repeat CT scan to rule out further hemorrhage showed no change from the previous in terms of hemorrhage, swelling or brain stem compression. A neurologic exam performed immediately after the CT scan, 30 minutes after the mannitol bolus, the patient had deteriorated with absent pupillary response, absent corneal reflexes and a total lack of eye movement on oculocephalic reflex testing, no spontaneous breathing and extensor posturing in the extremities to painful stimuli. It was felt that the patient was not salvageable. Before calling in the family and withdrawing active care, a transcranial Doppler (TCD) was performed in the emergency department to assess cerebral flow. The TCD was performed by the first author (a neurosurgeon and a neuro-intensivest), using a P4-1c Phased Array probe (ZONARE Medical Systems, Inc., Mountain View, CA, USA), operated at 2–3 mHz to insonate the temporal windows bilaterally. There was reverberating flow in the left middle cerebral artery (MCA) compatible with cerebral circulatory arrest on that side, but a high resistance pattern of flow in the right MCA (Bellner et al. 2004). Optic nerve ultrasonography was also performed and showed bilateral dilatation of the optic nerve sheath compatible with intracranial hypertension (Soldatos et al. 2009) (Figure 2).

**Figure 3:**
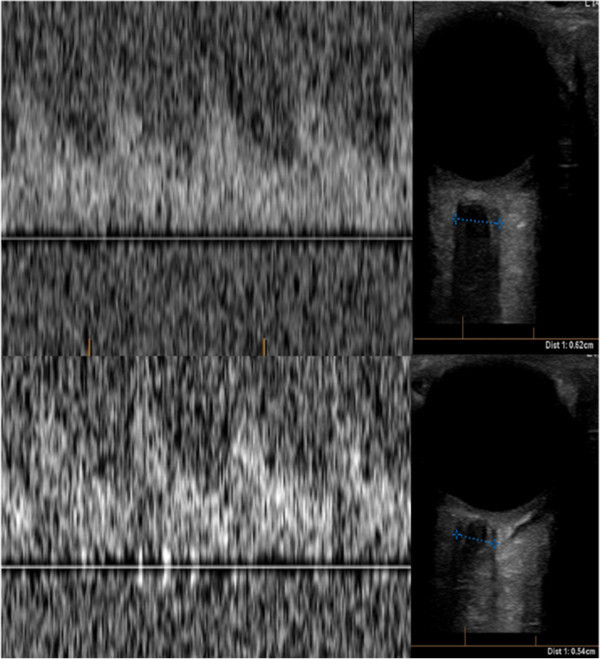
**This is the post mannitol and hyperventilation transcranial Doppler showing normal tracing in both middle cerebral arteries (Left upper and lower panels) with PI of 2.9 bilaterally.** In addition, there was a reduction of the diameter of the optic nerve sheath with the left and right side measuring 6.2 mm and 5.4 mm, respectively.

Because of the presence of flow demonstrated in the right MCA, the patient was given another 500 mL of 20% mannitol (1.53 g/kg) over five minutes with close monitoring of his blood pressure. Within a few minutes the TCD examination showed the return of circulation in both hemispheres along with a reduction in the diameter of the optic nerve sheaths bilaterally (Figure 3). Immediately after the TCD the patient was examined and had reactive pupils. Several minutes later he was localizing to pain with his left side. Based on this favorable response, the patient was rushed to the operating room for a decompressive craniectomy and expansive duraplasty along with evacuation of the hematoma and tissue sampling of the hemorrhagic mass. The patient was observed in the intensive care unit for a few days and then transferred to the neurosurgical ward awaiting further treatment for his lesion (biopsy revealed a glioblastoma). Upon transfer to a rehabilitation center, he still had significant right-sided weakness and dysphasia. He had no residual brain stem dysfunction and repeat imaging showed resolving hemorrhage and residual tumor as expected, as well as a left posterior cerebral artery ischemic stroke likely from the herniation syndrome the patient sustained at the beginning of his hospital course.

## Discussion

This case shows the potential utility of TCD examination in acute neurological deterioration to assess the extent of cerebral circulation compromise, the potential for reversal of circulatory arrest and the efficacy of the chosen dose of hyperosmolar therapy. In this case, the patient had no signs of brain stem function. This would have placed him on a conservative palliative approach given the poor neurological prognosis associated with such condition. The TCD on the other hand showed severely compromised yet persistent cerebral flow in a patient who was otherwise considered unsalvageable. This finding promted the use of an additional large dose of mannitol to objectively assess any changes in the cerebral blood flow dynamics. The TCD done after the mannitol dose demonstrated a tangible effect of hyperosmolar therapy on the cerebral circulation that was later responsible for a reversal of the herniation and the return of brain function. The patient care shifted from a consideration of a palliative approach to aggressive surgical and medical therapy with good outcome of the acute care of this patient, not achievable otherwise, had the TCD examination not been performed. This technique may also be useful in gauging whether the chosen dose of hyperosmotic agent is actually the appropriate one. In a pilot study by Tazarourte et al., TCD examination was carried out in a pre-hospital setting, or upon arrival, and patients were treated according to the TCD results of improved blood flow (Tazarourte et al. 2011). TCD was used to assess the efficacy of the maneuvers, and those patients for whom the cerebral perfusion could be corrected according to the pulsatility index fared better (Bellner et al. 2004).

Both TCD and optic nerve ultrasonography can be done with portable machines in the emergency department, intensive care unit and even in the operating room if necessary (Soldatos et al. 2009;Raboel et al. 2012). Performance of TCD examination by a trained technician or a physician is not time consuming. Most TCD units are easily transported to the point of care where the patient is (e.g. The emergency department or the operating room) and newer models even offer portable hand held forms of the device. The learning curve is not discouragingly steep. The major limitation is the logistic constrains of having a trained TCD performer available to capture such patients at these extreme conditions. In conclusion, the use of TCD and optic nerve ultrasonography at the point of care on patients with acute neurological deterioration could be a useful and objective adjunct to help gauge ICP and guide therapy outside of the Intensive Care environment and without the potential delays associated with the installation of invasive monitoring techniques.

## Conclusion

The use of transcranial Doppler in patients presenting with acute neurological deterioration could be a valuable tool to objectively and non-invasively assesses the intracranial pressure dynamics and guide effective course of treatment.

## Consent

Written informed consent was obtained from the patient for the publication of this report and any accompanying images.
